# Supplementary material for: The causal relationship between the human gut microbiota and pyogenic arthritis: a Mendelian randomization study
Source: Front Cell Infect Microbiol. 2024 Nov 26;14:1452480. doi: 10.3389/fcimb.2024.1452480 (PMC11629706; doi:10.3389/fcimb.2024.1452480)
Supplement: Supplementary file 1 [file DataSheet1.zip › mendelian test/ebi-a-GCST90027777.csv_forest.pdf]

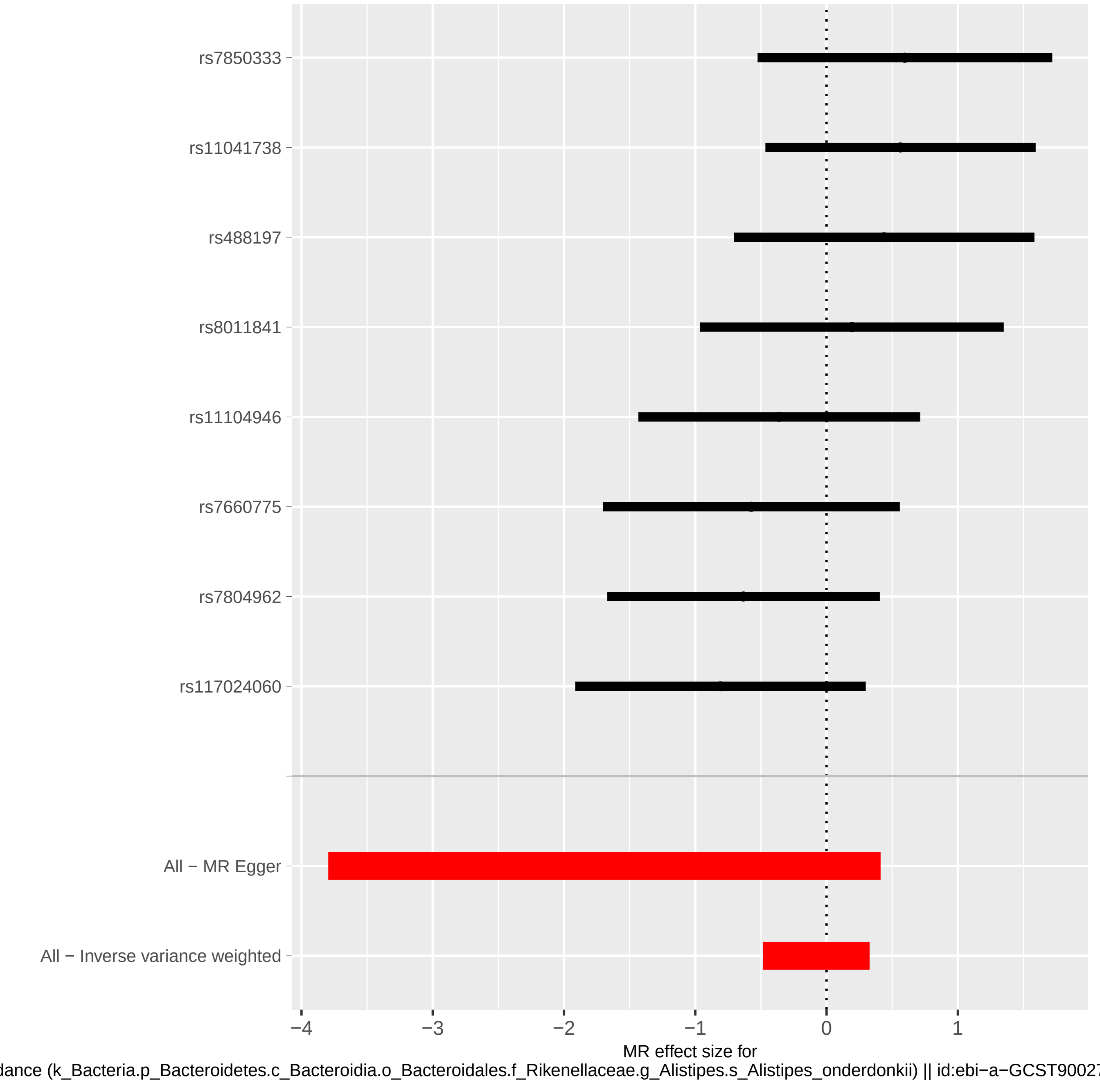

rs7850333
rs11041738
rs488197
rs8011841
rs11104946
rs7660775
rs7804962
rs117024060
All − MR Egger
All − Inverse variance weighted
−4
−3
−2
−1
0
1
MR effect size for
dance (k_Bacteria.p_Bacteroidetes.c_Bacteroidia.o_Bacteroidales.f_Rikenellaceae.g_Alistipes.s_Alistipes_onderdonkii) || id:ebi−a−GCST9002
